# [(Di-*o*-tolyl­phosphino)meth­yl]diphenyl­phosphine sulfide

**DOI:** 10.1107/S1600536809034059

**Published:** 2009-09-05

**Authors:** Priyanka P. Pitroda, Ansonia H. Badgett, Geralyn A. Dickey, Danielle L. Gray, Quinetta D. Shelby

**Affiliations:** a DePaul University, Department of Chemistry, 1110 West Belden Avenue, Chicago, Illinois 60614, USA; b University of Illinois, School of Chemical Sciences, Box 59-1, 505 South Mathews Avenue, Urbana, Illinois 61801, USA

## Abstract

In the title compound, C_27_H_26_P_2_S, the P—C—P angle is 114.33 (13)°. The bond distances are longer and the bond angles are smaller at the P atom bonded to the *o*-tolyl groups owing to the presence of a lone pair of electrons. One phenyl ring is disordered over three sites [occupancies 0.317 (8), 0.250 (8), and 0.433 (6)] and the other phenyl ring is disordered over two sites [occupancies 0.871 (6) and 0.129 (6)].

## Related literature

For the synthesis of unsymmetrical (phosphinometh­yl)phosphine monosulfides, see: Grim & Mitchell (1977 ▶); Grim *et al.* (1980 ▶). For the structures of related disulfides, see: Carmalt *et al.* (1996 ▶); Jones *et al.* (2002 ▶).
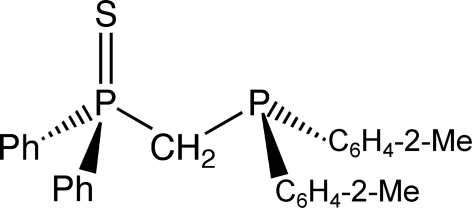


## Experimental

### 

#### Crystal data

C_27_H_26_P_2_S
*M* *_r_* = 444.48
Monoclinic, 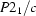

*a* = 20.0639 (15) Å
*b* = 7.2739 (5) Å
*c* = 16.4160 (11) Å
β = 92.519 (4)°
*V* = 2393.5 (3) Å^3^
*Z* = 4
Mo *K*α radiation
μ = 0.28 mm^−1^
*T* = 193 K
0.35 × 0.33 × 0.19 mm

#### Data collection

Bruker Kappa APEXII CCD diffractometer
Absorption correction: multi-scan (*SADABS*; Bruker, 2007 ▶) *T*_min_ = 0.935, *T*_max_ = 0.974
40516 measured reflections
4447 independent reflections
3092 reflections with *I* > 2σ(*I*)
*R*_int_ = 0.063

#### Refinement

*R*[*F* ^2^ > 2σ(*F* ^2^)] = 0.045
*wR*(*F* ^2^) = 0.112
*S* = 1.01
4447 reflections
439 parameters
713 restraints
H-atom parameters not refined
Δρ_max_ = 0.36 e Å^−3^
Δρ_min_ = −0.22 e Å^−3^

### 

Data collection: *APEX2* (Bruker, 2004 ▶); cell refinement: *SAINT* (Bruker, 2005 ▶); data reduction: *SAINT*; program(s) used to solve structure: *SHELXS97* (Sheldrick, 2008 ▶); program(s) used to refine structure: *SHELXL97* (Sheldrick, 2008 ▶); molecular graphics: *SHELXTL* (Sheldrick, 2008 ▶) and *CrystalMaker* (*CrystalMaker*, 1994 ▶); software used to prepare material for publication: *XCIF* (Bruker, 2005 ▶).

## Supplementary Material

Crystal structure: contains datablocks I, global. DOI: 10.1107/S1600536809034059/ng2633sup1.cif

Structure factors: contains datablocks I. DOI: 10.1107/S1600536809034059/ng2633Isup2.hkl

Additional supplementary materials:  crystallographic information; 3D view; checkCIF report

## Figures and Tables

**Fig. 1.**
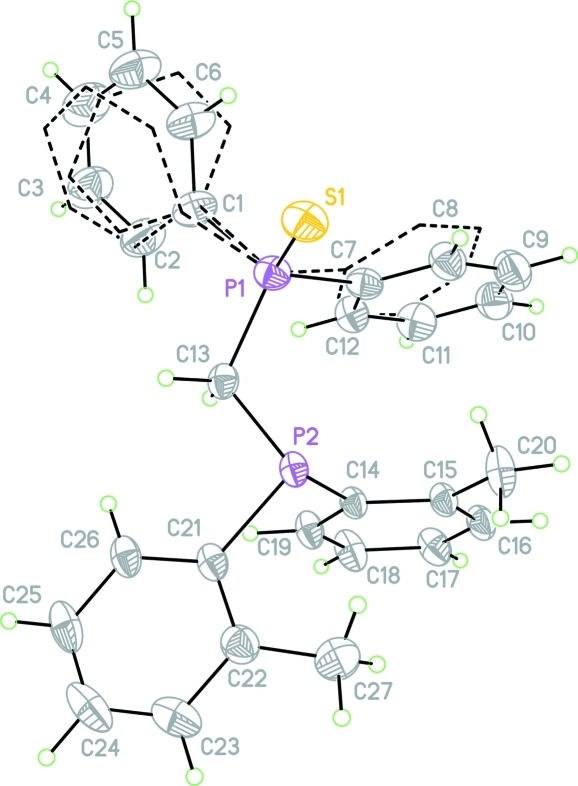
Molecular structure of the title compound showing disorder of the phenyl ring C1 to C6 over three sites and the phenyl ring C7 to C12 over two sites with 35% probability ellipsoids for non-H atoms and circles of arbitrary size for H atoms.

## supplementary crystallographic information

### Comment

Unsymmetrical (phosphinomethyl)phosphine monosulfides have been examined as ligands and have been used as precursors to their corresponding mixed bisphosphine ligands (Grim & Mitchell, 1977; Grim *et al.*, 1980). We are interested in the steric properties of mixed aryl-aryl bisphosphine sulfides and their derivatives. The title compound, (di-*o*-tolylphosphino)methyldiphenylphosphine sulfide (C_27_H_26_P_2_S), has not previously been reported, and its structure is shown in Fig. 1. The phenyl ring C1 to C6 is disordered over three sites and the phenyl ring C7 to C12 is disordered over two sites. The compound has a P1═S1 bond length of 1.950 (1) Å, which is similar to the P═S bond distances found in (bisphosphino)methane disulfides (Jones *et al.*, 2002; Camalt *et al.*, 1996). The P1—*C*(methylene) bond distance of 1.812 (3) Å is slightly shorter than the P2—*C*(methylene) bond distance of 1.852 (3) Å, where P1 is the diphenylphosphino P atom bonded to sulfur and P2 is the (di-*o*-tolyl)phosphino P atom. The P—C—P bond angle of 114.33 (13)° is larger than the expected value of 109.5° for a tetrahedral C atom. The C—P1—C bond angles range from 104.5 (4) to 106.15 (13)°. However, the C—P2—C bond angles of 100.23 (10) to 100.94 (11)° are significantly smaller. The longer bond distances and smaller bond angles at P2 are due to its lone pair of electrons.

### Experimental

The title compound was prepared from a procedure adapted from that described by Grim *et al.* (1980) for the synthesis of unsymmetric (phosphinomethyl)phosphine sulfides. Under an N_2_ atmosphere, Ph_2_PSCH_2_Li was formed from the addition of MeLi (8.84 ml of a 1.6 *M* solution in Et_2_O, 14.1 mmol) over 1 h to a suspension of Ph_3_PS (4.16 g, 14.1 mmol) in THF (16 ml) and Et_2_O (12 ml). After stirring an additional hour, the Ph_2_PSCH_2_Li solution was added to a suspension of (*o*-tolyl)_2_PCl (3.51 g, 14.1 mmol) in Et_2_O (16 ml) over 3 h. The mixture was stirred overnight. Solvents were removed under vacuum, and the residue was dissolved in CH_2_Cl_2_ (24 ml), washed with H_2_O (3 × 25 ml), and dried over MgSO_4_. Solvent was removed, and the resulting oil was dissolved in absolute EtOH (50 ml) to give colorless clusters of the title compound (2.69 g, 43%). Mp: 128.4–129.0 °C. Anal. Calcd for C_27_H_26_P_2_S: C, 72.96; H, 5.90; P, 13.94; S, 7.21. Found: C, 72.65; H, 5.94; P, 14.70; S, 7.28. ^1^H NMR (CDCl_3_): δ 2.30 (s, C*H*_3_), 3.34 (d, 12.5 Hz, C*H*_2_), 7.05–7.88 (m, C_6_*H*_5_ and C_6_*H*_4_CH_3_). ^13^C{^1^H} NMR (CDCl_3_): δ 21.3 (d, ^3^*J*_CP_ = 22 Hz, *C*H_3_), 33.1 (dd, ^1^*J*_CP_ = 54 Hz, ^1^*J*_CP_ = 32 Hz, *C*H_2_), 126.0 (s, *C*_6_H_4_CH_3_), 128.4 (d, *J* = 12 Hz, *m*-*C*_6_H_5_), 128.8 (s, *C*_6_H_4_CH_3_), 130.2 (d, *J* = 5 Hz, *C*_6_H_4_CH_3_), 131.2–131.7 (*o*-,*p*-*C*_6_H_5_ and *C*_6_H_4_CH_3_), 132.3 (d, ^1^*J*_CP_ = 82 Hz, *i*–P*C*_6_H_5_), 136.2 (dd, ^1^*J*_CP_ = 15,8 Hz, *i*–P*C*_6_H_4_CH_3_), 142.3 (d, ^2^*J*_CP_ = 29 Hz, *i*–*C*CH_3_). ^31^P {^1^H} NMR (CDCl_3_): δ -48.5 (d, ^2^*J*_PP_ = 74 Hz, *P*(C_6_H_4_CH_3_)_2_), 40.2 (d, ^2^*J*_PP_ = 74 Hz, *P*S(C_6_H_5_)_2_). IR (nujol mull between KBr plates, cm^-1^): C—H (3074, 3050), CC (1593, 1470, 1436), PS (745).

Single crystals suitable for X-ray diffraction were grown from slow diffusion of pentane into a concentrated EtOH solution at room temperature.

### Refinement

A structural model consisting of the molecule was developed. Two of the phenyl rings had poorly determined positions. In each disordered phenyl the geometry was idealized by restraining opposite C—C bond distances across the immaginary mirror plane that goes through the pivot carbon and C4' atom in the ring to be similar distances (e.s.d. 0.01). The C—P bond distances were restrained as similar distances (e.s.d. 0.01) and all phenyl rings in the disordered sites were forced to be flat (e.s.d. 0.01). The phenyl ring that contains atoms C1 thru C6 was disordered over 3 sites with each orientation being occupied by 31.7 (8), 25.0 (8), and 43.3 (6)% respectively. The phenyl ring that contains C7 thru C12 was disordered over two sites with the primary orientation being occupied 87.1 (6)% of the time. Rigid-bond restraints (e.s.d. 0.01) were imposed on displacement parameters for all disordered sites and similar displacement amplitudes (e.s.d. 0.01) were imposed on disordered sites overlapping by less than the sum of the Van der Waals radii. Methyl H atom positions, R—CH_3_, were optimized by rotation about R—C bonds with idealized C—H, R—H and H···H distances. Remaining H atoms were included as riding idealized contributors. Methyl H atom U's were assigned as 1.5 times *U*_eq_ of the carrier atom; remaining H atom U's were assigned as 1.2 times carrier *U*_eq_.

### Crystal data

**Table:**

C_27_H_26_P_2_S	*F*(000) = 936
*M**_r_* = 444.48	*D*_x_ = 1.233 Mg m^−^^3^
Monoclinic, *P*2_1_/*c*	Mo *K*α radiation, λ = 0.71073 Å
Hall symbol: -P 2ybc	Cell parameters from 5909 reflections
*a* = 20.0639 (15) Å	θ = 2.7–24.2°
*b* = 7.2739 (5) Å	µ = 0.28 mm^−^^1^
*c* = 16.4160 (11) Å	*T* = 193 K
β = 92.519 (4)°	Prism, colourless
*V* = 2393.5 (3) Å^3^	0.35 × 0.33 × 0.19 mm
*Z* = 4	

### Data collection

**Table:**

Bruker Kappa APEXII CCD diffractometer	4447 independent reflections
Radiation source: fine-focus sealed tube	3092 reflections with *I* > 2σ(*I*)
graphite	*R*_int_ = 0.063
profile data from φ and ω scans	θ_max_ = 25.6°, θ_min_ = 2.0°
Absorption correction: multi-scan (*SADABS*; Bruker, 2007)	*h* = −24→24
*T*_min_ = 0.935, *T*_max_ = 0.974	*k* = −8→8
40516 measured reflections	*l* = −19→19

### Refinement

**Table:**

Refinement on *F*^2^	Primary atom site location: structure-invariant direct methods
Least-squares matrix: full	Secondary atom site location: difference Fourier map
*R*[*F*^2^ > 2σ(*F*^2^)] = 0.045	Hydrogen site location: inferred from neighbouring sites
*wR*(*F*^2^) = 0.112	H-atom parameters not refined
*S* = 1.01	*w* = 1/[σ^2^(*F*_o_^2^) + (0.0481*P*)^2^ + 1.3507*P*] where *P* = (*F*_o_^2^ + 2*F*_c_^2^)/3
4447 reflections	(Δ/σ)_max_ = 0.001
439 parameters	Δρ_max_ = 0.36 e Å^−^^3^
713 restraints	Δρ_min_ = −0.22 e Å^−^^3^

### Special details

**Table:**

Experimental. One distinct cell was identified using *APEX2* (Bruker, 2004). Six frame series were integrated and filtered for statistical outliers using *SAINT* (Bruker, 2005) then corrected for absorption by integration using *SHELXTL*/*XPREP* V2005/2 (Bruker, 2005) before using *SADABS* (Bruker, 2005) to sort, merge, and scale the combined data. No decay correction was applied.
Geometry. All e.s.d.'s (except the e.s.d. in the dihedral angle between two l.s. planes) are estimated using the full covariance matrix. The cell e.s.d.'s are taken into account individually in the estimation of e.s.d.'s in distances, angles and torsion angles; correlations between e.s.d.'s in cell parameters are only used when they are defined by crystal symmetry. An approximate (isotropic) treatment of cell e.s.d.'s is used for estimating e.s.d.'s involving l.s. planes.
Refinement. Structure was phased by direct methods (Sheldrick, 2008). Systematic conditions suggested the unambiguous space group. The space group choice was confirmed by successful convergence of the full-matrix least-squares refinement on *F*^2^. The highest peaks in the final difference Fourier map were in the vicinity of atoms P1, P2, and C20; the final map had no other significant features. A final analysis of variance between observed and calculated structure factors showed little dependence on amplitude or resolution.

### Fractional atomic coordinates and isotropic or equivalent isotropic displacement parameters (Å^2^)

**Table:**

	*x*	*y*	*z*	*U*_iso_*/*U*_eq_	Occ. (<1)
S1	0.18814 (4)	−0.05273 (10)	0.82663 (4)	0.0549 (2)	
P1	0.17515 (4)	0.14683 (10)	0.90362 (4)	0.0423 (2)	
P2	0.32375 (3)	0.25293 (8)	0.91684 (4)	0.03177 (17)	
C1	0.0953 (4)	0.2626 (17)	0.8887 (9)	0.0559 (19)	0.317 (8)
C2	0.0797 (6)	0.4395 (18)	0.9115 (9)	0.071 (2)	0.317 (8)
H2A	0.1137	0.5115	0.9380	0.085*	0.317 (8)
C3	0.0176 (6)	0.5177 (16)	0.8982 (8)	0.078 (2)	0.317 (8)
H3A	0.0086	0.6398	0.9151	0.093*	0.317 (8)
C4	−0.0306 (6)	0.412 (2)	0.8595 (9)	0.087 (2)	0.317 (8)
H4A	−0.0738	0.4624	0.8487	0.104*	0.317 (8)
C5	−0.0178 (5)	0.235 (2)	0.8360 (10)	0.080 (2)	0.317 (8)
H5A	−0.0521	0.1646	0.8095	0.096*	0.317 (8)
C6	0.0448 (5)	0.1597 (17)	0.8507 (10)	0.067 (2)	0.317 (8)
H6A	0.0532	0.0367	0.8347	0.081*	0.317 (8)
C1B	0.0989 (6)	0.276 (2)	0.8738 (11)	0.061 (2)	0.250 (8)
C2B	0.0880 (7)	0.453 (2)	0.9018 (12)	0.068 (2)	0.250 (8)
H2BA	0.1211	0.5052	0.9379	0.082*	0.250 (8)
C3B	0.0327 (7)	0.558 (2)	0.8813 (10)	0.080 (2)	0.250 (8)
H3BA	0.0289	0.6783	0.9032	0.096*	0.250 (8)
C4B	−0.0169 (7)	0.490 (2)	0.8293 (10)	0.083 (2)	0.250 (8)
H4BA	−0.0552	0.5615	0.8147	0.100*	0.250 (8)
C5B	−0.0092 (6)	0.316 (2)	0.7994 (10)	0.086 (2)	0.250 (8)
H5BA	−0.0427	0.2648	0.7636	0.104*	0.250 (8)
C6B	0.0469 (6)	0.216 (2)	0.8210 (10)	0.071 (2)	0.250 (8)
H6BA	0.0506	0.0964	0.7984	0.085*	0.250 (8)
C1C	0.0946 (3)	0.2509 (10)	0.8906 (4)	0.0525 (16)	0.433 (6)
C2C	0.0866 (4)	0.4292 (11)	0.8658 (5)	0.0660 (17)	0.433 (6)
H2CA	0.1242	0.5051	0.8574	0.079*	0.433 (6)
C3C	0.0224 (4)	0.4969 (13)	0.8530 (5)	0.0841 (19)	0.433 (6)
H3CA	0.0154	0.6204	0.8359	0.101*	0.433 (6)
C4C	−0.0306 (4)	0.3838 (12)	0.8654 (5)	0.0897 (19)	0.433 (6)
H4CA	−0.0744	0.4310	0.8559	0.108*	0.433 (6)
C5C	−0.0232 (3)	0.2051 (12)	0.8908 (6)	0.083 (2)	0.433 (6)
H5CA	−0.0610	0.1300	0.8995	0.100*	0.433 (6)
C6C	0.0405 (3)	0.1378 (11)	0.9032 (5)	0.0685 (19)	0.433 (6)
H6CA	0.0474	0.0143	0.9204	0.082*	0.433 (6)
C7	0.17930 (17)	0.0688 (5)	1.00919 (18)	0.0414 (9)	0.871 (6)
C8	0.19203 (18)	−0.1139 (4)	1.0286 (2)	0.0522 (10)	0.871 (6)
H8A	0.1983	−0.2005	0.9863	0.063*	0.871 (6)
C9	0.1956 (2)	−0.1711 (5)	1.1092 (2)	0.0640 (12)	0.871 (6)
H9A	0.2044	−0.2964	1.1220	0.077*	0.871 (6)
C10	0.1867 (2)	−0.0475 (5)	1.1703 (2)	0.0607 (11)	0.871 (6)
H10A	0.1892	−0.0869	1.2255	0.073*	0.871 (6)
C11	0.1740 (2)	0.1334 (6)	1.1521 (2)	0.0565 (11)	0.871 (6)
H11A	0.1677	0.2189	1.1949	0.068*	0.871 (6)
C12	0.1704 (2)	0.1923 (5)	1.0719 (2)	0.0462 (9)	0.871 (6)
H12A	0.1618	0.3180	1.0598	0.055*	0.871 (6)
C7B	0.1592 (12)	0.102 (3)	1.0092 (7)	0.046 (3)	0.129 (6)
C8B	0.1518 (12)	−0.082 (3)	1.0271 (10)	0.052 (3)	0.129 (6)
H8BA	0.1495	−0.1707	0.9848	0.062*	0.129 (6)
C9B	0.1479 (13)	−0.135 (3)	1.1080 (10)	0.061 (2)	0.129 (6)
H9BA	0.1429	−0.2607	1.1214	0.073*	0.129 (6)
C10B	0.1512 (12)	−0.005 (3)	1.1684 (10)	0.060 (3)	0.129 (6)
H10B	0.1484	−0.0429	1.2236	0.072*	0.129 (6)
C11B	0.1585 (15)	0.177 (3)	1.1507 (12)	0.053 (3)	0.129 (6)
H11B	0.1606	0.2653	1.1934	0.064*	0.129 (6)
C12B	0.1626 (16)	0.232 (3)	1.0703 (13)	0.048 (3)	0.129 (6)
H12B	0.1678	0.3587	1.0575	0.058*	0.129 (6)
C13	0.23640 (12)	0.3288 (3)	0.89744 (14)	0.0368 (6)	
H13A	0.2261	0.4254	0.9375	0.044*	
H13B	0.2322	0.3845	0.8424	0.044*	
C14	0.33277 (12)	0.2801 (3)	1.02845 (13)	0.0319 (5)	
C15	0.35109 (13)	0.1284 (3)	1.07706 (14)	0.0355 (6)	
C16	0.35666 (13)	0.1529 (4)	1.16115 (15)	0.0436 (7)	
H16A	0.3688	0.0513	1.1950	0.052*	
C17	0.34508 (15)	0.3198 (4)	1.19610 (15)	0.0480 (7)	
H17A	0.3490	0.3328	1.2537	0.058*	
C18	0.32772 (15)	0.4695 (4)	1.14798 (15)	0.0506 (8)	
H18A	0.3196	0.5856	1.1721	0.061*	
C19	0.32229 (13)	0.4484 (3)	1.06448 (14)	0.0399 (6)	
H19A	0.3111	0.5516	1.0312	0.048*	
C20	0.36521 (17)	−0.0566 (4)	1.04103 (17)	0.0573 (8)	
H20A	0.3286	−0.0909	1.0025	0.086*	
H20B	0.3692	−0.1484	1.0847	0.086*	
H20C	0.4070	−0.0512	1.0124	0.086*	
C21	0.36910 (13)	0.4573 (3)	0.88391 (13)	0.0349 (6)	
C22	0.43854 (14)	0.4437 (4)	0.88096 (16)	0.0482 (7)	
C23	0.47409 (17)	0.5932 (5)	0.8522 (2)	0.0655 (9)	
H23A	0.5212	0.5849	0.8500	0.079*	
C24	0.4426 (2)	0.7518 (4)	0.82709 (18)	0.0670 (10)	
H24A	0.4678	0.8514	0.8070	0.080*	
C25	0.37510 (19)	0.7663 (4)	0.83110 (15)	0.0559 (8)	
H25A	0.3531	0.8768	0.8148	0.067*	
C26	0.33887 (15)	0.6207 (3)	0.85878 (14)	0.0415 (6)	
H26A	0.2918	0.6320	0.8608	0.050*	
C27	0.47553 (16)	0.2744 (5)	0.9088 (2)	0.0741 (10)	
H27A	0.4531	0.1654	0.8856	0.111*	
H27B	0.4763	0.2674	0.9684	0.111*	
H27C	0.5214	0.2797	0.8906	0.111*	

### Atomic displacement parameters (Å^2^)

**Table:**

	*U*^11^	*U*^22^	*U*^33^	*U*^12^	*U*^13^	*U*^23^
S1	0.0666 (5)	0.0505 (4)	0.0476 (4)	−0.0067 (4)	0.0020 (4)	−0.0170 (3)
P1	0.0467 (4)	0.0439 (4)	0.0360 (4)	−0.0044 (3)	0.0011 (3)	−0.0062 (3)
P2	0.0453 (4)	0.0260 (3)	0.0240 (3)	0.0012 (3)	0.0009 (3)	−0.0007 (2)
C1	0.040 (3)	0.071 (3)	0.056 (4)	0.000 (3)	0.001 (3)	−0.010 (3)
C2	0.052 (3)	0.075 (3)	0.084 (4)	0.007 (3)	−0.012 (4)	−0.009 (4)
C3	0.053 (4)	0.086 (4)	0.094 (4)	0.011 (3)	−0.004 (4)	−0.003 (4)
C4	0.051 (3)	0.099 (4)	0.110 (4)	0.014 (3)	−0.007 (4)	−0.001 (4)
C5	0.046 (3)	0.097 (4)	0.097 (5)	−0.001 (4)	−0.007 (4)	−0.008 (4)
C6	0.046 (3)	0.081 (4)	0.075 (5)	−0.003 (3)	−0.003 (4)	−0.011 (4)
C1B	0.043 (3)	0.075 (4)	0.064 (4)	−0.001 (3)	0.001 (3)	−0.013 (4)
C2B	0.050 (3)	0.075 (4)	0.078 (4)	0.009 (3)	−0.010 (4)	−0.008 (4)
C3B	0.053 (4)	0.089 (4)	0.096 (5)	0.009 (4)	−0.013 (4)	−0.006 (4)
C4B	0.049 (4)	0.095 (4)	0.105 (5)	0.007 (4)	−0.011 (4)	0.001 (4)
C5B	0.056 (4)	0.101 (5)	0.101 (5)	0.010 (4)	−0.012 (4)	−0.007 (4)
C6B	0.048 (4)	0.086 (4)	0.077 (5)	0.001 (3)	−0.003 (4)	−0.016 (4)
C1C	0.040 (3)	0.068 (3)	0.050 (3)	0.001 (3)	0.001 (3)	−0.010 (3)
C2C	0.048 (3)	0.080 (3)	0.071 (4)	0.014 (3)	0.000 (3)	0.000 (3)
C3C	0.059 (3)	0.090 (4)	0.102 (4)	0.014 (3)	−0.014 (4)	−0.001 (3)
C4C	0.051 (3)	0.102 (4)	0.115 (4)	0.019 (3)	−0.006 (3)	0.001 (4)
C5C	0.043 (3)	0.096 (4)	0.109 (5)	0.003 (3)	−0.007 (4)	0.001 (4)
C6C	0.045 (3)	0.078 (3)	0.082 (4)	0.002 (3)	−0.007 (3)	−0.003 (3)
C7	0.039 (2)	0.0454 (18)	0.0400 (16)	−0.0063 (15)	0.0060 (14)	−0.0034 (14)
C8	0.061 (3)	0.0448 (17)	0.0503 (19)	−0.0038 (17)	0.0025 (18)	−0.0024 (15)
C9	0.077 (3)	0.054 (2)	0.060 (2)	−0.009 (2)	0.000 (2)	0.0094 (17)
C10	0.065 (3)	0.068 (2)	0.0488 (19)	−0.013 (2)	0.0013 (18)	0.0137 (17)
C11	0.064 (3)	0.065 (2)	0.0410 (18)	−0.0041 (19)	0.0078 (17)	−0.0046 (17)
C12	0.053 (2)	0.0448 (19)	0.0410 (17)	0.0009 (17)	0.0072 (15)	0.0000 (15)
C7B	0.051 (6)	0.046 (5)	0.042 (5)	−0.005 (5)	0.008 (5)	−0.005 (4)
C8B	0.057 (6)	0.051 (5)	0.047 (4)	−0.003 (5)	0.007 (5)	−0.002 (5)
C9B	0.070 (5)	0.059 (4)	0.055 (4)	−0.008 (5)	0.005 (5)	0.007 (4)
C10B	0.068 (6)	0.065 (5)	0.048 (5)	−0.002 (5)	0.009 (5)	0.003 (4)
C11B	0.059 (6)	0.058 (5)	0.044 (5)	0.001 (5)	0.009 (5)	−0.003 (5)
C12B	0.052 (5)	0.051 (5)	0.041 (4)	0.000 (5)	0.009 (5)	−0.006 (4)
C13	0.0462 (16)	0.0366 (13)	0.0273 (13)	0.0028 (12)	−0.0012 (11)	−0.0007 (11)
C14	0.0413 (14)	0.0286 (12)	0.0258 (12)	−0.0027 (10)	0.0009 (11)	0.0035 (10)
C15	0.0463 (15)	0.0312 (12)	0.0290 (13)	−0.0029 (11)	0.0011 (11)	0.0056 (10)
C16	0.0578 (18)	0.0384 (14)	0.0341 (15)	−0.0083 (13)	−0.0032 (12)	0.0119 (12)
C17	0.075 (2)	0.0459 (16)	0.0225 (13)	−0.0131 (14)	0.0001 (13)	0.0025 (12)
C18	0.088 (2)	0.0335 (14)	0.0309 (15)	−0.0065 (14)	0.0042 (14)	−0.0035 (11)
C19	0.0645 (18)	0.0290 (12)	0.0261 (13)	−0.0012 (12)	0.0006 (12)	0.0000 (10)
C20	0.090 (2)	0.0353 (14)	0.0467 (17)	0.0146 (15)	0.0050 (16)	0.0089 (13)
C21	0.0525 (17)	0.0326 (13)	0.0197 (12)	−0.0025 (12)	0.0021 (11)	−0.0010 (10)
C22	0.0532 (19)	0.0486 (16)	0.0430 (16)	−0.0055 (14)	0.0026 (13)	0.0022 (13)
C23	0.065 (2)	0.066 (2)	0.067 (2)	−0.0211 (17)	0.0168 (17)	−0.0008 (17)
C24	0.105 (3)	0.0478 (18)	0.0497 (19)	−0.0274 (19)	0.0251 (19)	−0.0001 (15)
C25	0.103 (3)	0.0338 (15)	0.0310 (15)	−0.0033 (16)	0.0081 (16)	0.0023 (12)
C26	0.0657 (18)	0.0327 (13)	0.0261 (13)	0.0003 (12)	0.0022 (12)	0.0004 (10)
C27	0.0474 (19)	0.075 (2)	0.100 (3)	0.0043 (17)	0.0044 (18)	0.018 (2)

### Geometric parameters (Å, °)

**Table:**

S1—P1	1.950 (1)	C9—H9A	0.9500
P1—C1C	1.790 (5)	C10—C11	1.371 (4)
P1—C7B	1.806 (9)	C10—H10A	0.9500
P1—C13	1.812 (3)	C11—C12	1.385 (4)
P1—C1	1.816 (6)	C11—H11A	0.9500
P1—C7	1.822 (3)	C12—H12A	0.9500
P1—C1B	1.843 (7)	C7B—C12B	1.378 (8)
P2—C21	1.837 (2)	C7B—C8B	1.380 (8)
P2—C14	1.844 (2)	C8B—C9B	1.389 (8)
P2—C13	1.852 (3)	C8B—H8BA	0.9500
C1—C2	1.380 (7)	C9B—C10B	1.365 (8)
C1—C6	1.387 (7)	C9B—H9BA	0.9500
C2—C3	1.379 (7)	C10B—C11B	1.367 (8)
C2—H2A	0.9500	C10B—H10B	0.9500
C3—C4	1.369 (7)	C11B—C12B	1.386 (8)
C3—H3A	0.9500	C11B—H11B	0.9500
C4—C5	1.369 (7)	C12B—H12B	0.9500
C4—H4A	0.9500	C13—H13A	0.9900
C5—C6	1.383 (8)	C13—H13B	0.9900
C5—H5A	0.9500	C14—C19	1.380 (3)
C6—H6A	0.9500	C14—C15	1.401 (3)
C1B—C2B	1.386 (7)	C15—C16	1.391 (3)
C1B—C6B	1.397 (7)	C15—C20	1.502 (3)
C2B—C3B	1.377 (8)	C16—C17	1.367 (4)
C2B—H2BA	0.9500	C16—H16A	0.9500
C3B—C4B	1.375 (8)	C17—C18	1.381 (4)
C3B—H3BA	0.9500	C17—H17A	0.9500
C4B—C5B	1.367 (8)	C18—C19	1.379 (3)
C4B—H4BA	0.9500	C18—H18A	0.9500
C5B—C6B	1.376 (8)	C19—H19A	0.9500
C5B—H5BA	0.9500	C20—H20A	0.9800
C6B—H6BA	0.9500	C20—H20B	0.9800
C1C—C2C	1.367 (6)	C20—H20C	0.9800
C1C—C6C	1.384 (6)	C21—C26	1.388 (3)
C2C—C3C	1.385 (7)	C21—C22	1.400 (4)
C2C—H2CA	0.9500	C22—C23	1.394 (4)
C3C—C4C	1.367 (7)	C22—C27	1.499 (4)
C3C—H3CA	0.9500	C23—C24	1.370 (5)
C4C—C5C	1.371 (7)	C23—H23A	0.9500
C4C—H4CA	0.9500	C24—C25	1.363 (4)
C5C—C6C	1.376 (7)	C24—H24A	0.9500
C5C—H5CA	0.9500	C25—C26	1.373 (4)
C6C—H6CA	0.9500	C25—H25A	0.9500
C7—C12	1.383 (4)	C26—H26A	0.9500
C7—C8	1.387 (4)	C27—H27A	0.9800
C8—C9	1.386 (4)	C27—H27B	0.9800
C8—H8A	0.9500	C27—H27C	0.9800
C9—C10	1.364 (4)		
			
C1C—P1—C7B	89.6 (8)	C8—C9—H9A	120.0
C1C—P1—C13	107.2 (3)	C9—C10—C11	120.1 (3)
C7B—P1—C13	109.5 (8)	C9—C10—H10A	119.9
C7B—P1—C1	91.1 (9)	C11—C10—H10A	119.9
C13—P1—C1	104.5 (4)	C10—C11—C12	120.4 (3)
C1C—P1—C7	104.4 (2)	C10—C11—H11A	119.8
C13—P1—C7	106.15 (13)	C12—C11—H11A	119.8
C1—P1—C7	106.0 (5)	C7—C12—C11	120.3 (3)
C7B—P1—C1B	99.6 (10)	C7—C12—H12A	119.9
C13—P1—C1B	99.7 (6)	C11—C12—H12A	119.9
C7—P1—C1B	114.5 (6)	C12B—C7B—C8B	120.8 (10)
C1C—P1—S1	112.6 (2)	C12B—C7B—P1	124.8 (14)
C7B—P1—S1	121.6 (7)	C8B—C7B—P1	113.8 (12)
C13—P1—S1	113.40 (9)	C7B—C8B—C9B	118.9 (10)
C1—P1—S1	113.6 (4)	C7B—C8B—H8BA	120.5
C7—P1—S1	112.50 (12)	C9B—C8B—H8BA	120.5
C1B—P1—S1	109.9 (6)	C10B—C9B—C8B	120.1 (11)
C21—P2—C14	100.23 (10)	C10B—C9B—H9BA	120.0
C21—P2—C13	100.70 (11)	C8B—C9B—H9BA	120.0
C14—P2—C13	100.94 (11)	C9B—C10B—C11B	121.1 (12)
C2—C1—C6	117.0 (7)	C9B—C10B—H10B	119.5
C2—C1—P1	127.3 (8)	C11B—C10B—H10B	119.5
C6—C1—P1	115.7 (8)	C10B—C11B—C12B	119.7 (11)
C3—C2—C1	123.8 (8)	C10B—C11B—H11B	120.2
C3—C2—H2A	118.1	C12B—C11B—H11B	120.2
C1—C2—H2A	118.1	C7B—C12B—C11B	119.4 (11)
C4—C3—C2	117.2 (8)	C7B—C12B—H12B	120.3
C4—C3—H3A	121.4	C11B—C12B—H12B	120.3
C2—C3—H3A	121.4	P1—C13—P2	114.33 (13)
C5—C4—C3	121.3 (9)	P1—C13—H13A	108.7
C5—C4—H4A	119.3	P2—C13—H13A	108.7
C3—C4—H4A	119.3	P1—C13—H13B	108.7
C4—C5—C6	120.3 (8)	P2—C13—H13B	108.7
C4—C5—H5A	119.8	H13A—C13—H13B	107.6
C6—C5—H5A	119.8	C19—C14—C15	119.7 (2)
C5—C6—C1	120.3 (8)	C19—C14—P2	120.73 (18)
C5—C6—H6A	119.9	C15—C14—P2	119.56 (18)
C1—C6—H6A	119.9	C16—C15—C14	118.2 (2)
C2B—C1B—C6B	111.8 (7)	C16—C15—C20	119.8 (2)
C2B—C1B—P1	121.8 (10)	C14—C15—C20	122.0 (2)
C6B—C1B—P1	126.4 (10)	C17—C16—C15	121.5 (2)
C3B—C2B—C1B	124.9 (9)	C17—C16—H16A	119.3
C3B—C2B—H2BA	117.6	C15—C16—H16A	119.3
C1B—C2B—H2BA	117.6	C16—C17—C18	120.2 (2)
C4B—C3B—C2B	120.4 (9)	C16—C17—H17A	119.9
C4B—C3B—H3BA	119.8	C18—C17—H17A	119.9
C2B—C3B—H3BA	119.8	C19—C18—C17	119.2 (2)
C5B—C4B—C3B	117.7 (9)	C19—C18—H18A	120.4
C5B—C4B—H4BA	121.1	C17—C18—H18A	120.4
C3B—C4B—H4BA	121.1	C18—C19—C14	121.2 (2)
C4B—C5B—C6B	120.1 (9)	C18—C19—H19A	119.4
C4B—C5B—H5BA	119.9	C14—C19—H19A	119.4
C6B—C5B—H5BA	119.9	C15—C20—H20A	109.5
C5B—C6B—C1B	125.1 (9)	C15—C20—H20B	109.5
C5B—C6B—H6BA	117.5	H20A—C20—H20B	109.5
C1B—C6B—H6BA	117.5	C15—C20—H20C	109.5
C2C—C1C—C6C	121.7 (6)	H20A—C20—H20C	109.5
C2C—C1C—P1	122.0 (6)	H20B—C20—H20C	109.5
C6C—C1C—P1	116.2 (5)	C26—C21—C22	118.2 (2)
C1C—C2C—C3C	118.6 (6)	C26—C21—P2	124.4 (2)
C1C—C2C—H2CA	120.7	C22—C21—P2	117.34 (19)
C3C—C2C—H2CA	120.7	C23—C22—C21	118.8 (3)
C4C—C3C—C2C	119.2 (7)	C23—C22—C27	119.4 (3)
C4C—C3C—H3CA	120.4	C21—C22—C27	121.9 (2)
C2C—C3C—H3CA	120.4	C24—C23—C22	121.5 (3)
C3C—C4C—C5C	122.7 (7)	C24—C23—H23A	119.2
C3C—C4C—H4CA	118.6	C22—C23—H23A	119.2
C5C—C4C—H4CA	118.6	C25—C24—C23	119.7 (3)
C4C—C5C—C6C	118.0 (7)	C25—C24—H24A	120.1
C4C—C5C—H5CA	121.0	C23—C24—H24A	120.1
C6C—C5C—H5CA	121.0	C24—C25—C26	119.9 (3)
C5C—C6C—C1C	119.7 (6)	C24—C25—H25A	120.1
C5C—C6C—H6CA	120.1	C26—C25—H25A	120.1
C1C—C6C—H6CA	120.1	C25—C26—C21	121.8 (3)
C12—C7—C8	118.7 (3)	C25—C26—H26A	119.1
C12—C7—P1	120.2 (3)	C21—C26—H26A	119.1
C8—C7—P1	121.2 (2)	C22—C27—H27A	109.5
C9—C8—C7	120.5 (3)	C22—C27—H27B	109.5
C9—C8—H8A	119.7	H27A—C27—H27B	109.5
C7—C8—H8A	119.7	C22—C27—H27C	109.5
C10—C9—C8	120.1 (3)	H27A—C27—H27C	109.5
C10—C9—H9A	120.0	H27B—C27—H27C	109.5
			
C1C—P1—C1—C2	135 (12)	C1—P1—C7—C8	126.1 (5)
C7B—P1—C1—C2	78.6 (10)	C1B—P1—C7—C8	127.8 (6)
C13—P1—C1—C2	−31.9 (8)	S1—P1—C7—C8	1.4 (2)
C7—P1—C1—C2	80.0 (8)	C12—C7—C8—C9	0.1 (2)
C1B—P1—C1—C2	−89 (6)	P1—C7—C8—C9	179.4 (2)
S1—P1—C1—C2	−156.0 (6)	C7—C8—C9—C10	0.0 (2)
C1C—P1—C1—C6	−44 (11)	C8—C9—C10—C11	0.0 (4)
C7B—P1—C1—C6	−100.8 (11)	C9—C10—C11—C12	−0.2 (5)
C13—P1—C1—C6	148.7 (7)	C8—C7—C12—C11	−0.2 (4)
C7—P1—C1—C6	−99.4 (8)	P1—C7—C12—C11	−179.5 (3)
C1B—P1—C1—C6	91 (6)	C10—C11—C12—C7	0.2 (5)
S1—P1—C1—C6	24.6 (9)	C1C—P1—C7B—C12B	−77.1 (12)
C6—C1—C2—C3	−0.4 (3)	C13—P1—C7B—C12B	31.0 (13)
P1—C1—C2—C3	−179.9 (11)	C1—P1—C7B—C12B	−74.8 (13)
C1—C2—C3—C4	−0.4 (3)	C7—P1—C7B—C12B	110 (3)
C2—C3—C4—C5	0.8 (7)	C1B—P1—C7B—C12B	−73.0 (13)
C3—C4—C5—C6	−0.3 (9)	S1—P1—C7B—C12B	166.4 (11)
C4—C5—C6—C1	−0.6 (9)	C1C—P1—C7B—C8B	111.5 (13)
C2—C1—C6—C5	0.9 (6)	C13—P1—C7B—C8B	−140.4 (12)
P1—C1—C6—C5	−179.6 (9)	C1—P1—C7B—C8B	113.8 (14)
C1C—P1—C1B—C2B	93 (3)	C7—P1—C7B—C8B	−61 (3)
C7B—P1—C1B—C2B	70.6 (11)	C1B—P1—C7B—C8B	115.6 (14)
C13—P1—C1B—C2B	−41.3 (8)	S1—P1—C7B—C8B	−5.0 (17)
C1—P1—C1B—C2B	83 (6)	C12B—C7B—C8B—C9B	0.1 (3)
C7—P1—C1B—C2B	71.6 (8)	P1—C7B—C8B—C9B	171.9 (15)
S1—P1—C1B—C2B	−160.7 (6)	C7B—C8B—C9B—C10B	0.0 (3)
C1C—P1—C1B—C6B	−88 (3)	C8B—C9B—C10B—C11B	0.0 (7)
C7B—P1—C1B—C6B	−110.7 (14)	C9B—C10B—C11B—C12B	−0.1 (9)
C13—P1—C1B—C6B	137.4 (11)	C8B—C7B—C12B—C11B	−0.2 (7)
C1—P1—C1B—C6B	−99 (6)	P1—C7B—C12B—C11B	−171.0 (17)
C7—P1—C1B—C6B	−109.7 (11)	C10B—C11B—C12B—C7B	0.2 (9)
S1—P1—C1B—C6B	18.0 (14)	C1C—P1—C13—P2	175.8 (2)
C6B—C1B—C2B—C3B	0.3 (3)	C7B—P1—C13—P2	80.0 (8)
P1—C1B—C2B—C3B	179.2 (13)	C1—P1—C13—P2	176.5 (5)
C1B—C2B—C3B—C4B	0.0 (3)	C7—P1—C13—P2	64.67 (17)
C2B—C3B—C4B—C5B	0.1 (7)	C1B—P1—C13—P2	−176.1 (6)
C3B—C4B—C5B—C6B	−0.4 (9)	S1—P1—C13—P2	−59.34 (14)
C4B—C5B—C6B—C1B	0.8 (10)	C21—P2—C13—P1	168.44 (12)
C2B—C1B—C6B—C5B	−0.7 (7)	C14—P2—C13—P1	−88.82 (14)
P1—C1B—C6B—C5B	−179.5 (14)	C21—P2—C14—C19	45.2 (2)
C7B—P1—C1C—C2C	121.0 (9)	C13—P2—C14—C19	−57.9 (2)
C13—P1—C1C—C2C	10.7 (5)	C21—P2—C14—C15	−134.2 (2)
C1—P1—C1C—C2C	−3(12)	C13—P2—C14—C15	122.7 (2)
C7—P1—C1C—C2C	123.0 (5)	C19—C14—C15—C16	1.4 (4)
C1B—P1—C1C—C2C	−37 (3)	P2—C14—C15—C16	−179.21 (19)
S1—P1—C1C—C2C	−114.7 (4)	C19—C14—C15—C20	−178.1 (3)
C7B—P1—C1C—C6C	−62.1 (9)	P2—C14—C15—C20	1.3 (4)
C13—P1—C1C—C6C	−172.5 (4)	C14—C15—C16—C17	−0.4 (4)
C1—P1—C1C—C6C	174 (12)	C20—C15—C16—C17	179.1 (3)
C7—P1—C1C—C6C	−60.1 (5)	C15—C16—C17—C18	−0.3 (4)
C1B—P1—C1C—C6C	140 (3)	C16—C17—C18—C19	0.0 (4)
S1—P1—C1C—C6C	62.2 (5)	C17—C18—C19—C14	1.1 (4)
C6C—C1C—C2C—C3C	0.0 (3)	C15—C14—C19—C18	−1.8 (4)
P1—C1C—C2C—C3C	176.6 (5)	P2—C14—C19—C18	178.8 (2)
C1C—C2C—C3C—C4C	−0.2 (3)	C14—P2—C21—C26	−98.9 (2)
C2C—C3C—C4C—C5C	0.6 (6)	C13—P2—C21—C26	4.4 (2)
C3C—C4C—C5C—C6C	−0.8 (8)	C14—P2—C21—C22	83.8 (2)
C4C—C5C—C6C—C1C	0.5 (8)	C13—P2—C21—C22	−172.85 (19)
C2C—C1C—C6C—C5C	−0.1 (6)	C26—C21—C22—C23	−0.7 (4)
P1—C1C—C6C—C5C	−177.0 (5)	P2—C21—C22—C23	176.7 (2)
C1C—P1—C7—C12	−56.9 (3)	C26—C21—C22—C27	178.5 (3)
C7B—P1—C7—C12	−49 (3)	P2—C21—C22—C27	−4.1 (4)
C13—P1—C7—C12	56.2 (3)	C21—C22—C23—C24	0.0 (5)
C1—P1—C7—C12	−54.6 (5)	C27—C22—C23—C24	−179.1 (3)
C1B—P1—C7—C12	−52.8 (7)	C22—C23—C24—C25	0.9 (5)
S1—P1—C7—C12	−179.3 (2)	C23—C24—C25—C26	−1.2 (4)
C1C—P1—C7—C8	123.7 (3)	C24—C25—C26—C21	0.6 (4)
C7B—P1—C7—C8	132 (3)	C22—C21—C26—C25	0.4 (4)
C13—P1—C7—C8	−123.2 (2)	P2—C21—C26—C25	−176.84 (19)
